# Dopamine promotes *Klebsiella quasivariicola* proliferation and inflammatory response in the presence of macrophages

**DOI:** 10.3389/fcimb.2024.1322113

**Published:** 2024-03-22

**Authors:** Xiang Li, Lin Cheng, Xueyang Liu, Xiaoli Wang, Rui Li, Shao Fan, Qiulong Yan, Tonghui Ma, Yufang Ma, Jian Kang

**Affiliations:** ^1^ College of Basic Medical Sciences, Dalian Medical University, Dalian, China; ^2^ Department of Pathology, Changzhi People’s Hospital Affiliated to Changzhi Medical College, Changzhi, China; ^3^ Department of Oncology, Shanghai Medical College, Fudan University, Shanghai, China; ^4^ School of Medicine, Nanjing University of Chinese Medicine, Nanjing, China

**Keywords:** Klebsiella quasivariicola, dopamine, macrophages, inflammatory response, inflammatory injury

## Abstract

**Background:**

Dopamine, a frequently used therapeutic agent for critically ill patients, has been shown to be implicated in clinical infections recently, however, the precise mechanisms underlying this association remain elusive. *Klebsiella quasivariicola*, a novel strain belonging to the *Klebsiella* species, exhibits potential pathogenic attributes. The impact of dopamine on *K. quasivariicola* infection has aroused our interest.

**Objective:**

Considering the contribution of host immune factors during infection, this study aimed to investigate the intricate interactions between *K. quasivariicola*, dopamine, and macrophages were explored.

**Methods:**

RAW264.7 cells and C57/BL6 mice were infected with *K. quasivariicola*, and the bacterial growth within macrophage, the production of inflammatory cytokines and the pathological changes in mice lungs were detected, in the absence or presence of dopamine.

**Results:**

Dopamine inhibited the growth of *K. quasivariicola* in the medium, but promoted bacterial growth when co-cultured with macrophages. The expression of proinflammatory cytokines increased in RAW 264.7 cells infected with *K. quasivariicola*, and a significant rise was observed upon the addition of dopamine. The infection of *K. quasivariicola* in mice induced an inflammatory response and lung injury, which were exacerbated by the administration of dopamine.

**Conclusions:**

Our findings suggest that dopamine may be one of the potential risk factors associated with *K. quasivariicola* infection. This empirical insight provides solid references for clinical precision medicine. Furthermore, an *in vitro* model of microbes-drugs-host immune cells for inhibitor screening was proposed to more accurately replicate the complex *in vivo* environment. This fundamental work had contributed to the present understanding of the crosstalk between pathogen, dopamine and host immune cells.

## 1 Introduction

Nosocomial infections present a formidable challenge within intensive care units (ICU), leading to prolonged hospitalization, heightened risk of complications, and increased financial burdens on patients (Leone et al., 2018). The risk factors for nosocomial infection include the use of invasive treatments, the administration of hormones, and antibiotics (Shah et al., 2022; Yang et al., 2022). Notably, recent reports have raised concerns regarding the potential association between dopamine, a commonly administered drug in ICUs, and an increased risk of infection.

Dopamine (DA), a potent catecholamine vasopressor, has been widely used in various clinical settings, especially in antishock therapy to critically ill patients. However, recent clinical investigations have highlighted the relationship between dopamine and infections, prompting a cautious reassessment of its usage. A randomized controlled trial of pediatric septic shock showed that patients treated with dopamine had a higher infection rate compared to those treated with epinephrine (Ventura et al., 2015). Similarly, Hatachi et al. (Hatachi et al., 2018) reported that the administration of dopamine was a risk factor for infection in children following cardiac surgery. They indicated that the nosocomial infection rates were related to both the duration of dopamine administration and the total dose of dopamine. In addition, a retrospective study on extremely preterm infants demonstrated that the increased dopamine levels were associated with infection (Hotta et al., 2020). All of these findings suggest a potential correlation between dopamine treatment and infection; however, the underlying mechanism remains unknown.

A prospective observational study on nosocomial infections indicates that *Klebsiella* spp. ranked as the second most prevalent microorganisms in ICU patients suffering from secondary lower respiratory tract infections (Martin-Loeches et al., 2023). In our previous study, a metagenomic analysis was conducted to investigate the sputum microbiome in ICU patients with ventilator-associated pneumonia. The results showed a dramatic overgrowth of *Klebsiella* spp (Yan et al., 2016). Recently, using the culturomics approach, we successfully isolated a cohort of potentially pathogenic bacteria from sputum samples collected from ICU patients with pneumonia. It is worth noting that a substantial number of *Klebsiella* spp. were cultured. Among them a novel strain of *Klebsiella* species, *Klebsiella quasivariicola*, engrossed our attention.

*Klebsiella quasivariicola* was recently sequenced from a human clinical isolate (Long et al., 2017). Subsequently, *K. quasivariicola* strains were successively isolated from the wound infections of a patient with diabetic foot and also from urine samples of community-acquired infections, respectively (Garza-Ramos et al., 2018; Henciya et al., 2020). The whole-genome sequencing of *K. quasivariicola* revealed the presence of an extended-spectrum β-lactamase gene, indicating its potential to cause severe infections (Long et al., 2017). Further investigation demonstrated that, in addition to beta-lactam-resistant genes, fluoroquinolones-resistant genes and Colistin-resistant genes were also found in the *K. quasivariicola* genome (Uwanibe et al., 2023). Furthermore, studies have also confirmed that the *K. quasivariicola* strain is resistant to multiple drugs, including norfloxacin, ciprofloxacin, cefazolin, and vancomycin (Henciya et al., 2020). These findings indicate that *K. quasivariicola* is an important potential pathogen and should not be underestimated. In our previously study, we successfully identified *K. quasivariicola* in sputum samples obtained from pneumonia patients for the first time, so we speculate that *K. quasivariicola* could potentially act as a causative pathogen in pneumonia.

Relevant clinical observations have previously indicated a potential exacerbation of the infection with dopamine treatment. It is noteworthy that *K. quasivariicola* in our study was isolated from ICU patients with pneumonia who received dopamine treatment, we wondered whether dopamine affected the infection process of *K. quasivariicola* in patients. Therefore, the impacts of dopamine on the growth of *K. quasivariicola* were evaluated in this study. Furthermore, in order to simulate the process of infection in the host more accurately, immune factors were also taken into consideration. In this study, the effects of dopamine on *K. quasivariicola* were investigated using the RAW264.7 macrophage cell line and a mouse model, encompassing both growth dynamics and potential pathogenicity. Our findings revealed that dopamine inhibits the growth of *K. quasivariicola* in culture medium but promotes its viability in the presence of macrophages. The *K. quasivariicola* strain may induce pulmonary inflammation; however, administration of dopamine could potentiate the inflammatory response and exacerbate *K. quasivariicola*-induced infection. These insights shed light on the intricate crosstalk among pathogens, dopamine, and host immune cells. Significantly, our findings suggest that dopamine may serve as one of the potential risk factors associated with *K. quasivariicola* infection, providing a basis for clinical treatment.

## 2 Materials and methods

### 2.1 Bacterial strains and growth conditions

The *K. quasivariicola* strain used in this study was isolated from a sputum sample obtained from a pneumonia patient at the ICU of Central Hospital of Dalian University of Technology, Liaoning, China (formerly known as Dalian Municipal Central Hospital). The strain was identified through full-length sequencing of 16S rRNA gene (**Supplementary Figure S1**). The bacteria were cultured in DMEM (Gibco, United States) containing 10% FBS (Gibco, United States) at 37°C and supplemented with 500 µg/ml dopamine (Shanghai Harvest Pharmaceutical Co., Ltd, China) as required. The study was approved by the Ethics Committee of the Dalian Central Hospital (Ethical approval number: 2017-030-01). Informed consent was obtained from the subject. The clinical samples were collected in accordance with the approved guidelines.

For the growth of *K. quasivariicola* in cell culture supernatants, the RAW264.7 cells (2×10^6^) were cultured in DMEM containing 10% FBS and 500 μg/ml dopamine at 37°C. The cell cultures without dopamine were served as control. After 4 hours-cultivation, the supernatants were harvested and sterilized by passing it through a 0.22 μm filter (Millipore). The *K. quasivariicola* strain was then cultivated in supernatants at 37°C for 8 hours before CFU counting was done.

### 2.2 Bacterial growth with RAW264.7 macrophages

In 24-well plates, RAW264.7 cells (2×10^5^ per well) were seeded and cultured in DMEM containing 10% FBS and 500 ug/ml dopamine. The RAW264.7 control group was subjected to the same culture conditions as the experimental group, with the exception of the addition of dopamine. After culturing for 12 hours, RAW264.7 macrophages were infected with *K. quasivariicola* at a 10:1 MOI for 4 hours of incubation. The cell culture medium was then used to count the CFUs of *K. quasivariicola*. Meanwhile, RAW264.7 cells were collected and rinsed three times with PBS to remove extracellular bacteria. The cells were lysed with 500 μl of 0.03% SDS on ice. After homogenization, 10-fold serial dilutions were plated onto TSA plates to determine the CFU.

### 2.3 RNA isolation and RT-qPCR

RAW264.7 cells (4×10^5^ per well) were cultured in 12-well plates in DMEM containing 10% FBS. After ~12 hours, the cells were collected and resuspended in fresh DMEM medium containing 10% FBS. Then the cells were divided into 4 groups: RAW264.7 cells cultured in the absence of dopamine was considered as control group (CON), and the cells cultured with 500 μg/ml dopamine was considered as the dopamine group (DA). RAW264.7 cells infected with *K. quasivariicola* at an MOI of 10:1 was considered as K.q group (K.q), and the infected cells treated with 500 μg/ml dopamine was considered as Kq+DA group (K.q+DA). Cells were cultured for an additional 1, 4 or 12 hours, and then harvested for RNA isolation and RT-qPCR detection.

Total RNA of RAW264.7 cells were isolated using the RNAiso plus (TaKaRa, Japan) according to the manufacturer’s instructions. The isolated RNA (1 μg) was immediately reverse transcribed into cDNA using the AG Evo M-MLV RT Kit with gDNA Clean for qPCR Kit (Accurate Biotechnology, China). The expressions of TNF-α, IL-6, CXCL1, CXCL2, IFN-γ, β-actin, IL-8, iNOS, IL-17, IL-18 were analyzed by RT-qPCR using the specific primers list in **Table 1**. Data from three independent experiments were used for statistical analysis.

**Table 1:** Primers used for qPCR.

Gene	Primer sequence (5’-3’)
TNF-α F TNF-α R IL-6 F IL-6 R CXCL1 F CXCL1 R CXCL2 F CXCL2 R IFN-γ F IFN-γ R β-actin F β-actin R NLRP3 F NLRP3 R iNOS F iNOS R	ACTGAACTTCGGGGTGATCGGT TGGTTTGCTACGACGTGGGCTA CCCCAATTTCCAATGCTCTCC CGCACTAGGTTTGCCGAGTA ACCCAAACCGAAGTCATA AGGTGCCATCAGAGCAGT CCCAGACAGAAGTCATAGC TCCTTTCCAGGTCAGTTA CAGGCCATCAGCAACAACATAAGC AGCTGGTGGACCACTCGGATG TGACGTTGACATCCGTAAAGACC CTCAGGAGGAGCAATGATCTTGA ATCAACAGGCGAGACCTCTG GTCCTCCTGGCATACCATAGA GCTCGCTTTGCCACGGACGA AAGGCAGCGGGCACATGCAA

### 2.4 Immunofluorescence staining

Cells were fixed in 4% paraformaldehyde for 15 minutes at room temperature before being permeabilized in 0.1% Triton X-100 for 15 minutes. After rinsing with PBS, cells were blocked with 1% BSA for 60 min at room temperature. After blocking, cells were incubated with anti-NLRP3 antibody (Boster, Wuhan, China) overnight at 4°C. Cells were then washed with PBS to remove the excessive antibodies, and incubated with fluorescent secondary antibodies for 1 hour at 37°C. Samples were counterstained with DAPI (Invitrogen, Carlsbad, CA, United States) for 15 minutes to visualize the nuclei. Finally, the images were analyzed using an inverted fluorescent microscope (Olympus, Japan). The fluorescence intensity of NLRP3 was measured using Image J soft and the mean fluorescence intensity (MFI) was calculated using the following formula: MFI = ∑(Fluorescence intensity × Number of cells with that intensity)/Total number of cells.

### 2.5 Mouse model of acute lung injury

All animal experiments were approved by the Committee on the Ethics of Animal Experiments of Dalian Medical University (Permission number: SYXK (Liao) 2018-0007) and were performed in strict accordance with the recommendations. Sixteen C57/BL6 mice (aged 8 weeks, weight 20-25 g) were randomly divided into four experimental groups: (1) control group (CON), (2) dopamine group (DA), (3) *K. quasivariicola* group (k.q), and (4) *K. quasivariicola* + dopamine group (k.q+DA). In control group, the mice were intraperitoneal injection with saline. In dopamine group, mice were induced by intraperitoneal injections of same amount of 50 μg/g of dopamine. In *K. q* group, 1×10^8^ CFU of *K. quasivariicola* in 50 μl saline was administrated by oropharyngeal instillation. And in *K. quasivariicola* + dopamine group, in addition to *K. quasivariicola* infection, 50 μg/g of dopamine was also given to mice. For each group, a second administration were performed 24 hours later. All the mice were ready for the sacrifice after 48 hours of first treatment. The homogenates of lung tissue were used for RNA isolation and the expressions of TNF-α, IL-6, CXCL1, CXCL2, IFN-γ, β-actin, IL-8, iNOS, IL-17, IL-18 were analyzed by RT-qPCR as described previously.

### 2.6 Lung histopathology

The lung tissue was fixed in 4% paraformaldehyde, paraffin-embedded, and cut into slices for routine HE staining and immunohistochemical assays. Briefly, paraffin-embedded tissues were sliced into 4 μm thick slices, dewaxed and gradually rehydrated with ethanol. The sections were then treated for 20 minutes at 95°C with DAKO Target Retrieval solution (Dako) for epitope retrieval. Following that, the sections were treated with 0.3% hydrogen peroxide and probed overnight with anti-IL-6, anti-TNF-α and anti-NLRP3 primary antibodies. The secondary antibodies were then added and incubated for 30 minutes. The proteins were detected and visualized with streptavidin-HRP conjugates and DAB substrate solution. For HE staining, the deparaffinized sections were stained with hematoxylin, then treated with 1% acid alcohol, and finally with 1% eosin.

### 2.7 Statistical analysis

Comparisons of two groups were performed using unpaired Student’s t-test, and comparisons of multiple groups were performed using one-way analysis of variance (ANOVA). The data were analyzed using GraphPad Prism 7. The results were presented as means ± standard deviation (SD) from three independent experiments. A significant difference was defined as a *p*-value < 0.05.

## 3 Results

### 3.1 Dopamine promoted the proliferation of *K. quasivariicola* when bacteria were co-cultured with macrophages

**Figure 1:**
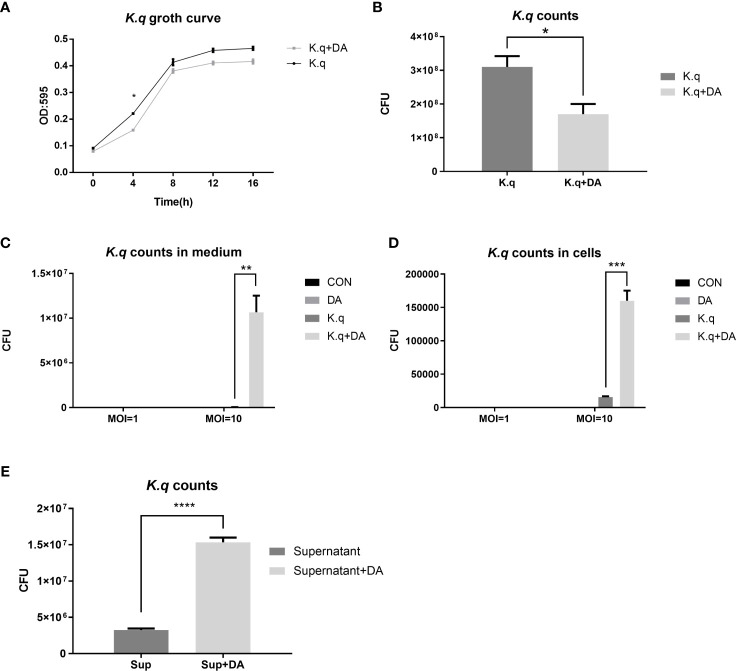
Growth curve and CFU count of *K. quasivariicola* under different conditions **(A, B)** The growth curve **(A)** and CFU counting **(B)** of *K. quasivariicola* in DMEM Basic medium. Black, *K*. *quasivariicola* cultured only; Gray, *K*. *quasivariicola* cultured with 500 µg/ml dopamine. **(C, D)**. After infecting RAW 264.7 cell with *K*. *quasivariicola*, CFU counts of *K*. *quasivariicola* in cell culture medium **(C)** and in macrophages **(D)** were performed. **(E)** CFU of *K*. *quasivariicola* grown in supernatant of RAW 264.7 culture medium. RAW264.7 cells were cultured in DMEM medium containing 10% FBS, in the absence (black) or presence (gray) of 500 μg/ml dopamine. The supernatants of cell culture medium were collected and used to cultivate *K*. *quasivariicola.* The CFU were determined after 8 hours of cultivation. The OD_595_ of cultures were monitored every 4 hours. Each sample was assayed in triplicate. The data are displayed as the mean ± standard error. *p*<0.05 was considered to indicate a statistically significant difference. *p<0.05, **p<0.01, ***p<0.001, ****p<0.0001.

To determine the impact of dopamine on *K. quasivariicola* growth, bacterial proliferation was measured in the presence of dopamine. When cultured in DMEM Basic medium for 4 hours, the growth of *K. quasivariicola* treated with 500 μg/ml dopamine exhibited a certain degree of decline (approximately a 1.8-fold decrease) compared to that of the bacterial culture without dopamine, *p*<0.05 (**Figures 1A, B**). However, after incubation with RAW264.7 for 4 h (MOI=10:1), the numbers of *K. quasivariicola* in the cell culture medium significantly increased when supplemented with 500 μg/ml dopamine (**Figure 1C**). Moreover, the presence of dopamine significantly increased the number of *K. quasivariicola* within macrophages, resulting in a tenfold amplification compared to bacterial culture without dopamine (**Figure 1D**). These findings suggest that dopamine can enhance the proliferation of *K. quasivariicola* in the presence of macrophages.

Furthermore, the supernatants of RAW 264.7 cultures were also collected for cultivation of *K. quasivariicola*. The RAW 264.7 cells were cultured in DMEM Basic medium containing 500 μg/ml dopamine for 4 hours, and then the culture supernatants (cell-free) were collected. Interestingly, the growth of *K. quasivariicola* in supernatants derived from dopamine-treated cells exhibited a 4.7-fold increase compared to bacterial growth in dopamine-minus supernatants (**Figure 1E**).

### 3.2 Dopamine significantly elevated the levels of pro-inflammatory factors in RAW 264.7 in response to *K. quasivariicola* infection

**Figure 2:**
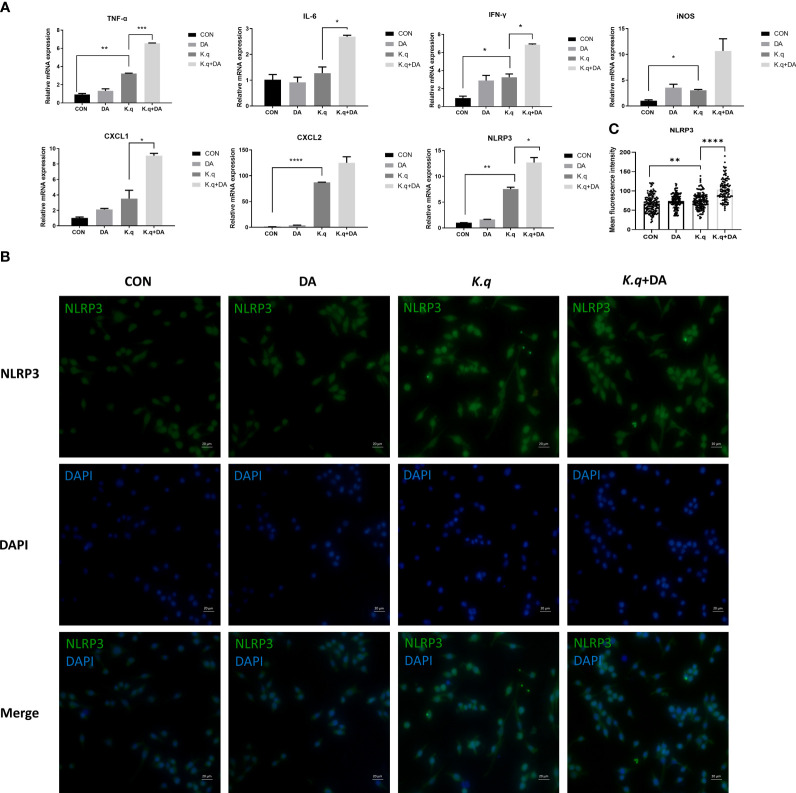
The detection of inflammatory factors in RAW 264.7 RAW264.7 were cultured in DMEM medium containing 10% FBS, in the absence (control guoup) or presence (dopamine group) of 500 μg/ml dopamine. RAW264.7 cells infected with *K*. *quasivariicola* at an MOI of 10:1 was considered as *K. q* group, and infected cells treated with 500 μg/ml dopamine was considered as K.q+DA group. After 4 hours of infection, the cytokines expressed in RAW264.7 cells were detected by qPCR **(A)** and immunofluorescence analysis **(B)**, respectively. **(C)** The histogram displays mean fluorescence intensity of NLRP3. Each sample was assayed in triplicate. The data are displayed as the mean ± standard error. *p*<0.05 was considered to indicate a statistically significant difference. *p<0.05, **p<0.01, ***p<0.001, ****p<0.0001.

To detect the inflammatory responses triggered by *K. quasivariicola*, a panel of cytokines produced by RAW 264.7 were measured after infection with *K. quasivariicola*. After 4 hours of *K. quasivariicola* infection, the mRNA levels of iNOS, IL-6, TNF-ɑ, IFN-γ, as well as the chemokines CXCL1, CXCL2, and the NLRP3 inflammasome were markedly increased (**Figure 2A**). The addition of dopamine in K.q+DA group further elevated mRNA levels of these pro-inflammatory cytokines by 1.5-3.5 times compared to the K.q group. Moreover, immunofluorescence co-localization analysis was utilized to visualize the NLRP3 protein. The results revealed an increase in NLRP3 expression in response to *K. quasivariicola* infection, which further rose when dopamine was added (K.q+DA) (**Figures 2B, C**). All these findings indicate that *K. quasivariicola* can induce the production of a range of pro-inflammatory factors in RAW 264.7, and the use of dopamine exacerbates inflammation.

### 3.3 *K. quasivariicola* led to the lung infection and proinflammatory response in mice, and dopamine made outcome of *K. quasivariicola* infection worsen

**Figure 3:**
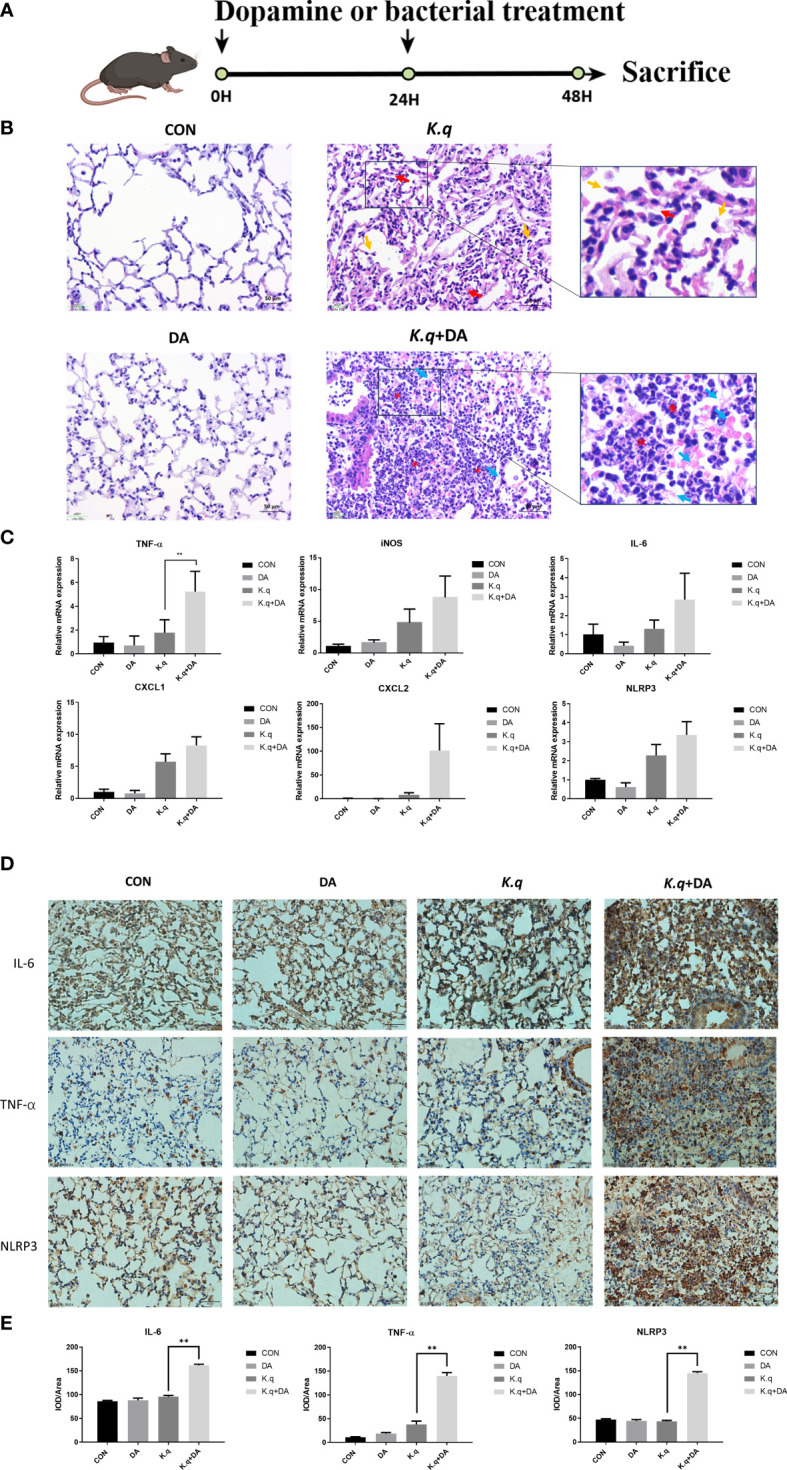
Lung histology of *K. quasivariicola* infected mice **(A)** The schematic overview of animal experimental design. **(B)** The mice were randomly divided into four groups: control group (CON), dopamine group (DA), *K*. *quasivariicola* group (K.q), and dopamine + *K*. *quasivariicola* group (K.q+DA). The CON group received a saline intraperitoneal injection. The DA group was induced by intraperitoneal injections of same amount of 50 μg/g of dopamine. In K.q group, 1×10^8^ CFU of *K*. *quasivariicola* in 50 μl saline was administrated by oropharyngeal instillation. And in the K.q+DA group, in addition to *K*. *quasivariicola* infection, 50 μg/g of dopamine was also given to mice. For each group, a second administration was performed 24 hours later. After 48 hours of treatment, lung tissues from each group were collected and stained with H&E. The red arrows represent alveolar collapse and the yellow arrows represent disruption of the alveolar septum. The blue arrows represent erythrocyte and fibrin diapedesis. The red asterisks indicate infiltration of inflammatory cells. The inflammatory factors in lung tissues from each group were detected. **(C)** The mRNA level of cytokines in mice lung. **(D)** The immunohistochemistry staining images of IL-6, TNF-α, and NLRP3 level in *in situ* lung tissue of mice. Brown and blue color in the image indicate positive staining of target protein and nuclei stained with hematoxylin, respectively. **(E)** The histogram displays the relative quantitative protein expression of IL-6, TNF-α, and NLRP3 in immunohistochemistry. Each sample was assayed in triplicate. The data are displayed as the mean ± standard error. *p*<0.05 was considered to indicate a statistically significant difference. *p<0.05, **p<0.01, ***p<0.001, ****p<0.0001.

A murine model of acute lung injury was utilized to assess the pathogenesis induced by *K. quasivariicola* (**Figure 3A**). The HE staining of lung tissue in *K. quasivariicola*-infected mice revealed disruption of alveolar structure, characterized by alveolar collapse (red arrows) and a broken alveolar septum (yellow arrows), thus suggesting the potential role of *K. quasivariicola* as a causative agent for pneumonia (**Figure 3B**). Although alveolar disruption was evident in mice from the K.q group, no significant infiltration of inflammatory cells was observed. However, in the K.q+DA group, a significant reduction of alveolar structures was observed, accompanied by a substantial infiltration of inflammatory cells (red asterisks) and exudation of erythrocyte and fibrinous material (blue arrows), indicating that dopamine administration exacerbated the inflammatory effects induced by *K. quasivariicola* (**Figure 3B**).

The expression of proinflammatory factors in C57/BL6 mice infected with *K. quasivariicola* were also measured. Consistent with the results from the cell experiment, we observed an upregulation of IL-6, iNOS, TNF-α, CXCL1, CXCL2 and NLRP3 in *K. quasivariicola*-infected mice. Moreover, dopamine further augmented their expression levels approximately 1.5 to 12.5-fold compared to the K.q group (**Figure 3C**). Additionally, the immunohistochemical staining revealed that the upregulation of IL-6, TNF-α, and NLRP3 proteins in *K. quasivariicola*-infected mice was not obvious; however, the administration of dopamine significantly increased their expressions, *p*<0.05 (**Figures 3D, E**).

## 4 Discussions

### 4.1 The crosstalk of dopamine and macrophage promoted the proliferation of *K. quasivariicola*

Dopamine, commonly utilized as a rescue drug in the ICU for shock management, had been shown to have intricate effects on bacterial growth. While some studies indicated inhibitory effects on bacterial growth, others showed growth stimulation (Li et al., 2015; Dichtl et al., 2019; Torabi Delshad et al., 2019; Choby et al., 2020). Our study extended this understanding by investigating the impact of dopamine on *K. quasivariicola* growth within a co-culture context with macrophages. While dopamine inhibited *K. quasivariicola* growth in culture medium (**Figures 1A, B**), its presence significantly amplified bacterial proliferation in the presence of macrophages (**Figures 1C, D**), suggesting a pivotal role of macrophages in mediating the interaction between dopamine and *K. quasivariicola*.

The same phenomenon was observed in *S. typhimurium*, where the bacterial load significantly increased upon the addition of dopamine during co-culture with macrophages. The research indicated that dopamine could promote the intracellular growth of *S. typhimurium* by augmenting iron acquisition (Dichtl et al., 2019). Iron was well known to be essential for bacterial growth, and the limitation of iron could impair the bacterial replication. One strategy bacterium employ to acquire iron is the synthesis of endogenous siderophores, which are iron-binding molecules used to capture and internalize ferric iron (Liu et al., 2014). It is interesting that a catechol core structure has been found in siderophores. As catecholamines share a catechol structure, they have been proven to transfer iron into bacteria, thereby promoting bacterial growth in low-iron media (Sandrini et al., 2010). Therefore, one potential mechanism by which dopamine facilitates the growth of *K. quasivariicola* is through enhancing bacterial iron uptake.

Additionally, the supernatants derived from RAW 264.7 cell cultures exhibited the ability to enhance *K. quasivariicola* growth, indicating the presence of a putative “growth-promoting factor” secreted by dopamine-induced macrophages. It is plausible that the potential growth factor in question could be attributed to the iron released from the macrophages from the macrophages. Further investigations are warranted to explore this intriguing avenue.

### 4.2 Dopamine triggered the exacerbation of inflammation response due to the facilitation of *K. quasivariicola* growth

To determine the pathogenic potential of *K. quasivariicola*, a mouse pneumoniae model by *K. quasivariicola* infection was established. Our findings revealed a significant upregulation of pro-inflammatory cytokines and the NLRP3 inflammasome in RAW 264.7 cells following a 4-hour infection with *K. quasivariicola*. Moreover, the addition of dopamine led to a subsequent increase (**Figure 2**). These compelling evidences indicate that *K. quasivariicola* infection can elicit a pulmonary inflammatory response, which is significantly exacerbated by the administration of dopamine.

Prior research has documented the anti-inflammatory properties of dopamine, as it facilitates the ubiquitinated degradation of NLRP3 inflammasome and consequently inhibits the inflammatory response (Yang et al., 2017; Feng and Lu, 2021). It was showed that dopamine can inhibit the LPS-induced activation of the NLRP3 inflammasome and reduce the subsequent production of caspase-1 and IL-1β (Yang et al., 2017). Further studies indicated that dopamine negatively regulates the NLRP3 inflammasome through the G protein pathway. The binding of dopamine to its receptor (D1-like receptor), a G protein-coupled receptor, stimulates the activity of adenylate cyclase and promotes the production of cAMP. cAMP can directly bind to NLRP3, triggering the ubiquitination of NLRP3 and leading to autophagy-mediated degradation of NLRP3 (Feng and Lu, 2021).

Contrary to previous reports, our results demonstrated that the administration of dopamine contributed to a more severe inflammation. The similar phenomenon has also been reported by Dicht et al (Dichtl et al., 2019), where *S. typhimurium i*nfected mice receiving dopamine showed a significantly increased immune response. Considering the anti-inflammatory properties of dopamine, we believed that during *K. quasivariicola* infection, dopamine does not directly interact with the immune system to aggravate the immune response. Based on our findings, we have deduced that dopamine triggers an intense inflammatory response by significantly promoting the proliferation of *K. quasivariicola*. Consequently, the substantial increase in bacterial load subsequently leads to a surge in the release of pro-inflammatory cytokines and aggravation of inflammation.

It’s worth noting that the influence of dopamine on immune response during *K. quasivariicola* infection appears to be indirect, focusing on bacterial load rather than direct immunomodulation. This suggests that the administration of dopamine to *K. quasivariicola*-infected patients may lead to an aggravated inflammation and a higher risk of cytokine storm. Therefore, for patients infected with *K. quasivariicola*, epinephrine or norepinephrine is considered a more favorable alternative to dopamine.

Nevertheless, certain limitations are evident in our study. Although we observed an increase in mRNA levels of inflammatory factors in the lungs of *K. quasivariicola*-infected mice, and the addition of dopamine further augmented this trend, there was no statistical significance. Enhancing the sample size has the potential to effectively address this issue.

### 4.3 *In vitro* model for inhibitor screening

**Figure 4:**
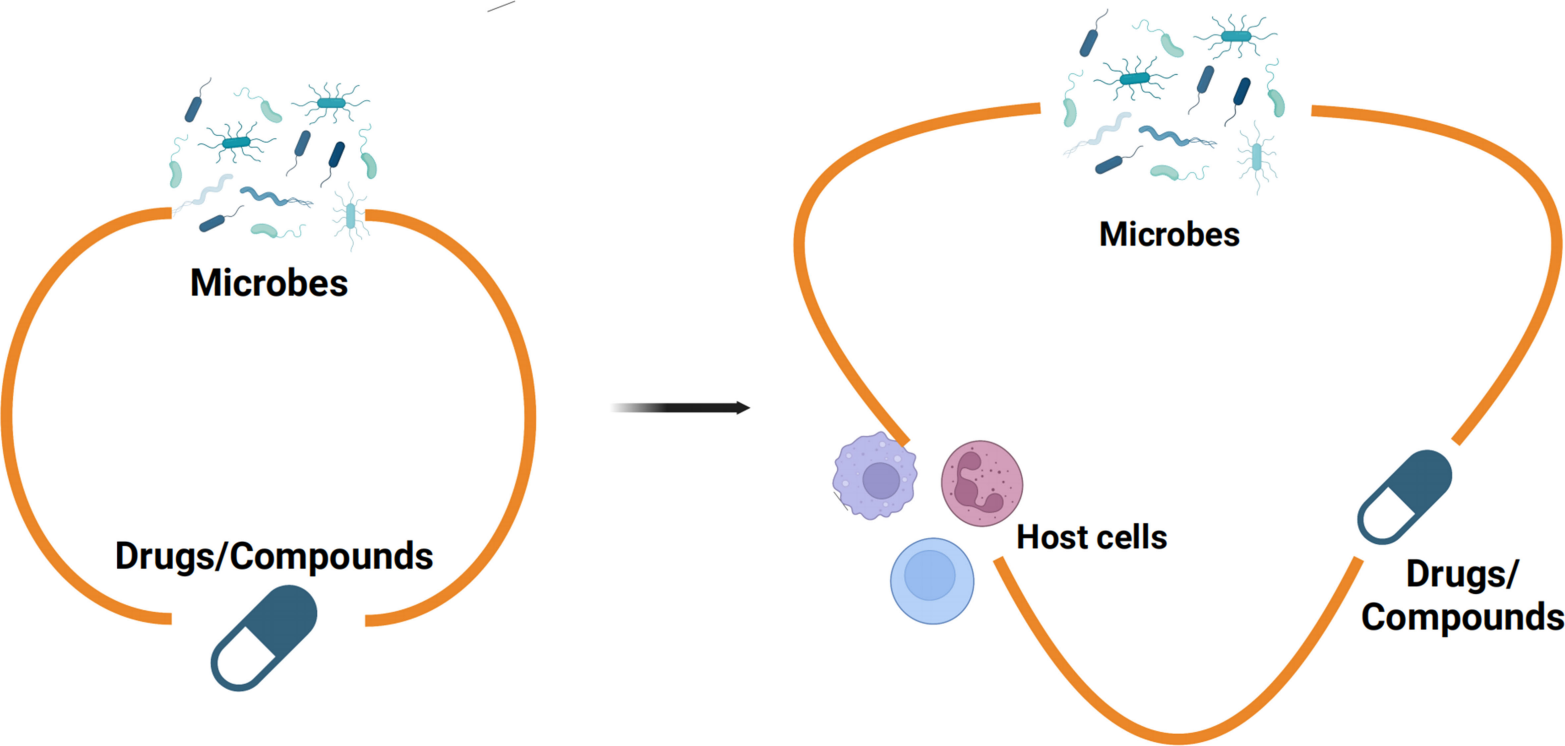
The *in vitro* model of microbes-drugs-host immune cells Previous studies on the interactions between microbes and monomeric compounds have predominantly relied on culture media, neglecting the influence of the host immune system (left). Nevertheless, there were more sophisticated communications between microbes and drugs in the niche of pathogens colonized in hosts. Therefore, an *in vitro* model which contains microbes-drugs-host immune cells was proposed for inhibitor screening (right). The *in vitro* model of microbes-drugs-host immune cells can incorporate host immune factors to more accurately simulate the living conditions of bacteria *in vivo*.

Our research highlights the dynamic interplay among dopamine, *K. quasivariicola*, and host immune cells, emphasizing the importance of an *in vitro* model that incorporates microbes, drugs, and host immune cells to better emulate *in vivo* conditions (**Figure 4**). By incorporating host immune responses, the proposed model would enhance assessments of microbial-drug interactions, thereby yielding more robust outcomes in inhibitor screening assays.

In conclusion, our study reveals that *K. quasivariicola* has the potential to be a causative agent of pneumonia, and the inflammatory effects of *K. quasivariicola* are further intensified by the action of dopamine. Dopamine may facilitate the growth of *K. quasivariicola* by promoting the transfer of iron into bacteria, leading to a substantial increase in bacterial load and subsequent upregulation of pro-inflammatory cytokine release, exacerbating inflammation. We suggested exercising caution when using dopamine for critical patients with *K. quasivariicola* infection, and recommended alternative catecholamines such as epinephrine or norepinephrine. Additionally, the *in vitro* model we proposed holds great potential for refining inhibitor screening and advancing comprehension of microbial-drug-host interactions.

## Data availability statement

The original contributions presented in the study are included in the article/**Supplementary Material**. Further inquiries can be directed to the corresponding authors.

## Ethics statement

The animal study was approved by Committee on the Ethics of Animal Experiments of Dalian Medical University (Permission number: SYXK (Liao) 2018-0007). The study was conducted in accordance with the local legislation and institutional requirements.

## Author contributions

XL: Conceptualization, Data curation, Investigation, Methodology, Visualization, Writing – original draft. LC: Conceptualization, Data curation, Investigation, Methodology, Visualization, Writing – original draft. XL: Conceptualization, Data curation, Investigation, Methodology, Visualization, Writing – original draft. XW: Investigation, Validation, Writing – original draft. RL: Data curation, Validation, Writing – original draft. SF: Data curation, Validation, Writing – original draft. QY: Funding acquisition, Validation, Visualization, Writing – original draft. TM: Conceptualization, Methodology, Resources, Supervision, Writing – review & editing. YM: Conceptualization, Methodology, Resources, Supervision, Writing – review & editing. JK: Conceptualization, Methodology, Project administration, Writing – original draft, Writing – review & editing, Funding acquisition.
