# Supplementary material for: Dopamine promotes Klebsiella quasivariicola proliferation and inflammatory response in the presence of macrophages
Source: Front Cell Infect Microbiol. 2024 Mar 22;14:1322113. doi: 10.3389/fcimb.2024.1322113 (PMC10995343; doi:10.3389/fcimb.2024.1322113)
Supplement: Supplementary file 1 [file DataSheet_1.docx]

**Supplementary Material**

Supplementary Figure 1. The 16S rRNA gene search in EzBioCloud Database and Colony morphology of *K. quasivariicola*

*
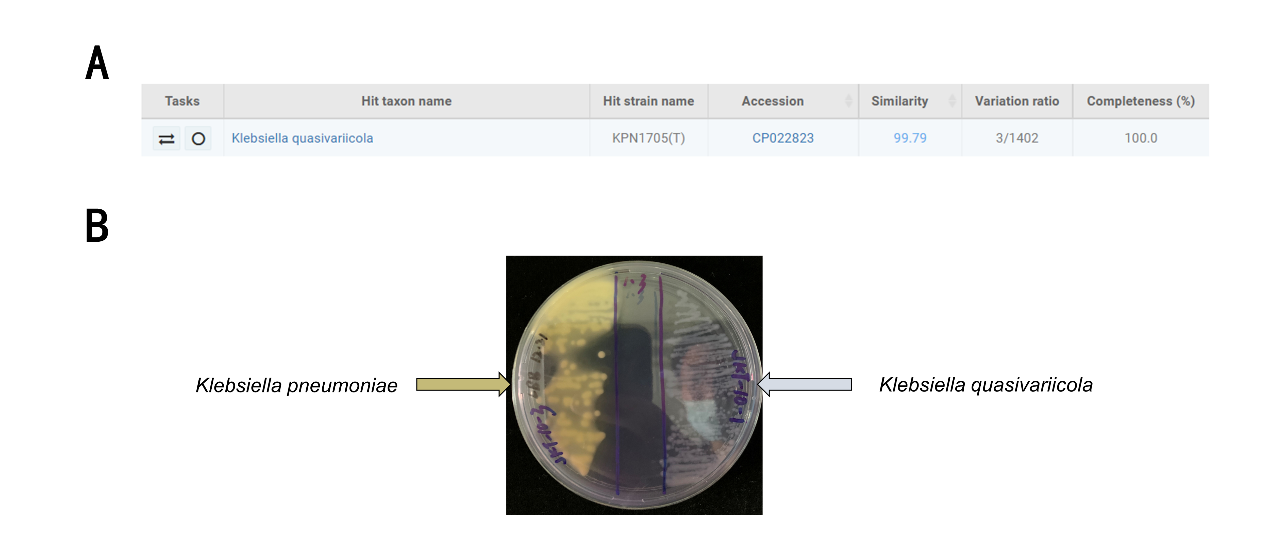
*
